# RNase J1 and J2 Are Host-Encoded Factors for Plasmid Replication

**DOI:** 10.3389/fmicb.2021.586886

**Published:** 2021-05-04

**Authors:** Vanessa Andrade Guimarães, Alexandre Le Scornet, Vanessa Khemici, Stéphane Hausmann, Joshua Armitano, Julien Prados, Ambre Jousselin, Caroline Manzano, Patrick Linder, Peter Redder

**Affiliations:** ^1^Department of Microbiology and Molecular Medicine, Faculty of Medicine, University of Geneva, Geneva, Switzerland; ^2^Laboratoire de Microbiologie et Génétique Moléculaires, Centre de Biologie Integrative, Paul Sabatier University, Toulouse, France

**Keywords:** *Staphylococcus aureus*, plasmid replication control, RNase J, antisense RNA, essential host factors

## Abstract

Plasmids need to ensure their transmission to both daughter-cells when their host divides, but should at the same time avoid overtaxing their hosts by directing excessive host-resources toward production of plasmid factors. Naturally occurring plasmids have therefore evolved regulatory mechanisms to restrict their copy-number in response to the volume of the cytoplasm. In many plasmid families, copy-number control is mediated by a small plasmid-specified RNA, which is continuously produced and rapidly degraded, to ensure that its concentration is proportional to the current plasmid copy-number. We show here that pSA564 from the RepA_N-family is regulated by a small antisense RNA (RNA1), which, when over-expressed *in trans*, blocks plasmid replication and cures the bacterial host. The 5′ untranslated region (5′UTR) of the plasmid replication initiation gene (*repA*) potentially forms two mutually exclusive secondary structures, ON and OFF, where the latter both sequesters the *repA* ribosome binding site and acts as a *rho*-independent transcriptional terminator. Duplex formation between RNA1 and the 5′UTR shifts the equilibrium to favor the putative OFF-structure, enabling a single small RNA to down-regulate *repA* expression at both transcriptional and translational levels. We further examine which sequence elements on the antisense RNA and on its 5′UTR target are needed for this regulation. Finally, we identify the host-encoded exoribonucleases RNase J1 and J2 as the enzymes responsible for rapidly degrading the replication-inhibiting section of RNA1. This region accumulates and blocks RepA expression in the absence of either RNase J1 or J2, which are therefore essential host factors for pSA564 replication in *Staphylococcus aureus*.

## Introduction

The bacterium *Staphylococcus aureus* is a versatile opportunistic pathogen present in the nasal cavities of 20–30% of the population (Mulcahy and McLoughlin, 2016). In some instances, *S. aureus* can cause life threatening diseases such as endocarditis, osteomyelitis, or sepsis, and it is well known for the production of several toxins (Lowy, 1998). A major problem of *S. aureus* infection is the frequent presence of antibiotic resistant strains which are difficult to eradicate. Indeed penicillin resistance had already been reported for *S. aureus* by the early 1940s (Lobanovska and Pilla, 2017), and *S. aureus* has been proven to be a champion in acquiring antibiotic resistances by horizontal gene transfer. This transfer is particularly efficient if the resistance gene is carried on a plasmid, as in the case of β-lactamases. Many different types of plasmids have been described for *S. aureus*, small and large, low and high copy number, using theta and rolling circle replication mechanisms, conjugative or not (Firth et al., 2018). The RepA_N family plasmids are generally large and replicate via a theta-replication mechanism. They usually encode a marker for penicillin resistance and frequently have acquired additional resistance cassettes as well. Although the RepA_N family is widespread among Firmicutes, each individual plasmid appears to have a quite narrow host range (Weaver et al., 2009).

All plasmids must regulate their copy number in order to limit the burden for the host cell. While several control mechanisms exist, most *S. aureus* plasmids described use an antisense RNAs for controlling replication initiation (Brantl, 2014). For example in pSK1 and pSK41 from the RepA_N plasmid family, the 5′UTR of *repA* can form two mutually exclusive structures, one of which (the OFF-structure) sequesters the *repA* ribosome binding site (RBS) and forms a putative *rho*-independent transcription terminator, whereas the other structure (the ON-structure) prevents the OFF-structure from forming (Kwong et al., 2006, 2008; Kwong and Firth, 2015). A plasmid-expressed antisense RNA can form a duplex with the *repA* 5′UTR and prevent formation of the ON-structure, thereby shifting the equilibrium toward the OFF-structure. Thus, increasing copy-number of the plasmid will increase the concentration of antisense RNA, which in turn will block replication initiation (Kwong et al., 2006, 2008; Weaver et al., 2009).

A prerequisite for regulation of gene expression by antisense RNAs is that the intra-cellular concentrations of these regulators tightly follow the concentration of the regulated plasmids. This is accomplished by strong constitutive expression of the asRNA combined with its rapid degradation (Pritchard et al., 1969), and the plasmid thus relies on an efficient host-encoded RNA decay machinery to correctly regulate its copy-number (Saramago et al., 2015). In *S. aureus*, the main enzymes in RNA degradation are the endoribonuclease RNase Y, the 5′–3′ exoribonuclease RNase J (RNase J1 and RNase J2), the 3′–5′ exoribonuclease PNPase, the double-stranded RNase III, and the RNA helicase CshA (Giraud et al., 2015; Durand and Condon, 2018).

*Staphylococcus aureus* strain SA564 (Somerville et al., 2002) was originally isolated from a patient with toxic shock syndrome (Somerville et al., 2002). In the course of sequencing its genome, we discovered that it carries a plasmid of 27273 bp, which we named pSA564 (Giraud et al., 2015). This previously uncharacterized plasmid carries a β-lactamase gene and encodes a replication initiator protein belonging to the RepA_N family (Weaver et al., 2009). Here we show that pSA564 replication is regulated by a small antisense RNA, RNA1, which controls the expression of RepA. We furthermore show that the level of RNA1 is regulated by RNase J-dependent degradation, and that RNA1 accumulates in RNase J mutant strains. This accumulation inhibits RepA production, thus making RNase J an essential host-factor for pSA564 replication. Since many plasmids are regulated by small unstable RNAs (Brantl, 2014), it is probable that this is an example of a general mechanism where RNase activities and specificities play an essential role in determining the host-range of plasmids.

## Results

Analysis of the pSA564 sequence revealed that it encodes a protein belonging to the RepA_N family of replication proteins (Weaver et al., 2009), between coordinates 20,793 to 21,737. The plasmid furthermore encodes a beta-lactamase, a cadmium transporter and three enterotoxin genes, as well as several hypothetical protein genes (Supplementary Figure 1).

**FIGURE 1:**
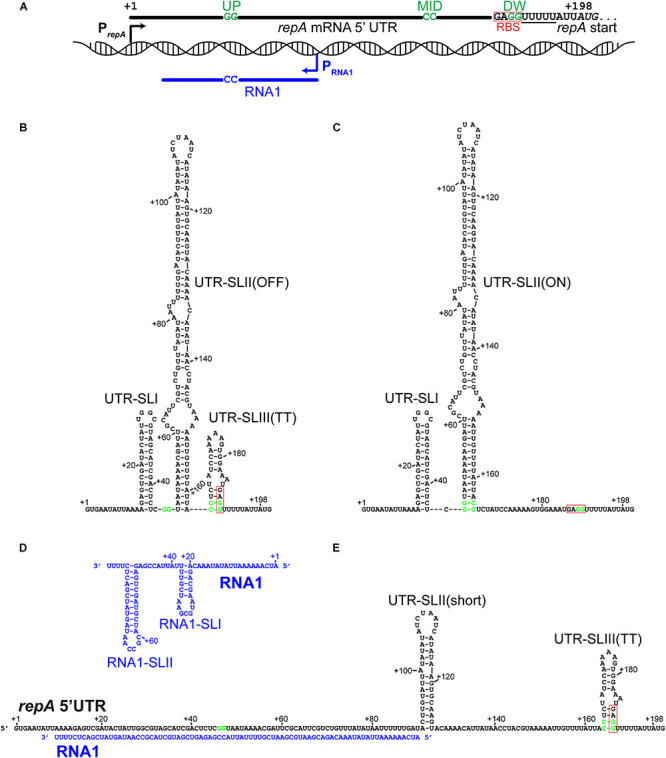
The 5′UTR of the *repA* mRNA can form putative regulatory structures that are modified by hybridization with the antisense RNA1. Fat lines indicate RNA, with key nucleotides shown. GG^UP^, CC^MID^, and GG^DW^ are indicated on the *repA* UTR RNA (in green) and the CC^*RNA*1^ complementary to GG^UP^ are indicated on RNA1. The poly-U stretches that are part of the putative rho-independent transcriptional termination structures are underlined. The Ribosome Binding Site (RBS) has been highlight with a red box. **(A)** Overview of the pSA564 genetic locus where the *repA* regulatory elements are found. The *repA* and RNA1 promoters are shown as black and blue arrows, respectively. RBS indicates the GAGG RBS. The *repA* start codon is in italics and the first nucleotide in the start codon is at position +198 of the transcript. **(B)** The proposed OFF-structure of the *repA* UTR, where CC^MID^ and GG^DW^ base-pairs in the UTR-SLIII(TT) stem-loop to form a rho-independent transcriptional terminator, and at the same time sequester the RBS. This structure is favored by pairing of the mRNA with the complementary RNA as shown in **(E)**. **(C)** The proposed ON-structure of the *repA* UTR, where GG^UP^ and CC^MID^ base-pairs in the UTR-SLII(ON) stem-loop, which prevents the formation of UTR-SLIII(TT) **(B)**. **(D)** The putative secondary structure of RNA1, where RNA1-SLII doubles as kissing loop and a transcription terminator. **(E)** RNA1 in a duplex along its full length with the *repA* UTR. UTR-SLI cannot form, the UTR-SLII stem is much shorter (only 14 base-pairs), but formation of UTR-SLIII(TT) is possible.

A GenBank analysis (performed in December 2019) showed that many *S. aureus* strains harbor plasmids highly similar to pSA564, with 683 sequenced plasmids encoding identical RepA_N proteins. More distantly related plasmids are also recognizably part of the same family; for example the pUSA300-HOU-MS and pSK1 RepA_N proteins are 99% and 68% identical to pSA564 RepA_N, respectively (Table 1). The *repA* transcription start site (TSS) was mapped to position 20,596, 197 nucleotides upstream of the AUG start codon (Prados et al., 2016). Although the nucleotide sequence of the 5′UTR from pSA564 *repA* is different from that of pSK1 and pSK41 (Kwong et al., 2006, 2008; Kwong and Firth, 2015), the *repA* mRNA 5′UTR also has the potential to form two mutually exclusive stem-loop structures, UTR-SLII-ON vs. UTR-SLII-OFF and UTR-SLIII (Figures 1B,C). These are distinguished by a central CC dinucleotide (CC^MID^) which can base-pair with either an upstream or downstream GG dinucleotide (GG^UP^ and GG^DW^, respectively). One of these two putative secondary structures sequesters the RBS of the *repA* gene while the other leaves it accessible to the ribosome (Figure 1).

**TABLE 1:** Similarities between RepA from pSA564 and RepA from other staphylococcal plasmids.

**Plasmid name**	**Identity (%)**	**Positive (%)**	RepA accession number*
pN315	45	64	BAB43870
pSK1	68	79	AAF63252
pUSA300-HOU-MR	45	63	YP_001569048
pUSA300-HOU-MS	99	99	YP_001569077

*The accession number for pSA564 RepA is ALD82318.

### pSA564 Is Regulated by a Small Antisense RNA Upstream of the *repA* Start Codon

**FIGURE 2:**
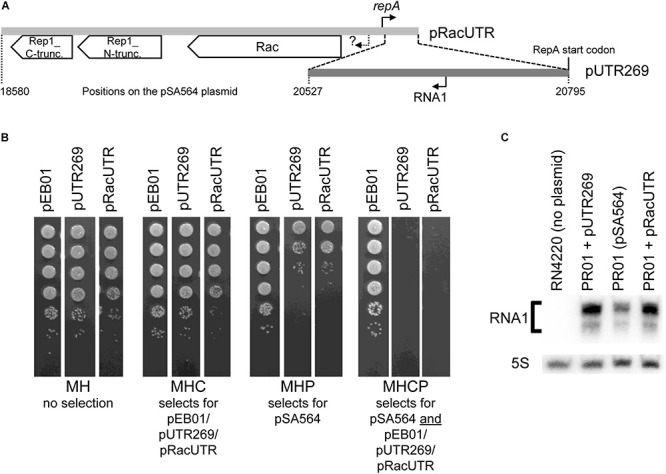
An incompatibility factor is encoded within 269 bp of pSA564. **(A)** The region upstream of the *repA* gene contains two divergent genes, *rac* and *rep1*, where the latter has been split in two (and presumably inactivated) by a frameshift mutation to form *rep1_N-truncation* and *rep1_C-truncation*. The 2,216 bp region cloned into pRacUTR (light gray), includes the putative *rac* promoter (question mark) and the *repA* promoter. The 269 bp region cloned into pUTR269 (dark gray) includes the *repA* start codon and the RNA1 promoter, but not the *repA* promoter. **(B)** pSA564 is lost upon acquisition of either pRacUTR or pUTR269. Colonies from the transformation were picked, sequentially diluted 10-fold and spotted on Mueller-Hinton plates containing either no antibiotic, chloramphenicol, penicillin G, or both antibiotics. **(C)** Northern blotting shows a small antisense RNA, transcribed from pSA564 as well as from pRacUTR and pUTR269. The minor band observed below the main RNA1 signal is presumably a fragment of RNA1, since it is absent from the *S. aureus* strain RN4220 which carries no plasmid. Probe R1 was used to detect RNA1, and a probe against 5S rRNA was used as control.

The region upstream of the *repA* gene contains two divergent genes, *rac* and *rep1*. The former could potentially contribute to plasmid segregation, while the latter appears to be a rolling circle plasmid replication protein which has been inactivated by a frameshift mutation (Figure 2A). To examine whether pSA564 replication is regulated by a small transcript (in the same way as pSK1 and pSK41), we generated two constructs using the multi-copy vector pEB01 as backbone. pEB01 carries a chloramphenicol resistance cassette and the pT181 origin for replication in *S. aureus* (Charpentier et al., 2004; Giraud et al., 2015). The first construct, pRacUTR, had an insert that contained a disrupted *rep_1* gene, the putative partitioning gene *rac*, as well as the promoter, 5′ untranslated region (5′UTR) and start codon of the *repA* gene (Figure 2A and Supplementary Figure 1). The second construct, pUTR269, had a 269 bp fragment containing the *repA* 5′UTR and start codon (Figure 2A and Supplementary Figure 1).

Strain PR01 is a derivative of SA564 (the natural host of pSA564). It carries pSA564 but the restriction systems have been knocked out to allow transformation with plasmids isolated from *E. coli*. PR01 was transformed with pRacUTR and pUTR269. Transformant colonies were resuspended in MH broth and plated on MH with chloramphenicol (MHC, to detect pRacUTR and pUTR269), MH with penicillin (MHP, to detect pSA564), and MH with both antibiotics (MHCP) (Figure 2B). The bacteria that received either pRacUTR or pUTR269 showed 2 orders of magnitude fewer colonies on penicillin plates, suggesting that the incoming pUTR269 or pRacUTR plasmids inhibited replication of the resident pSA564. In accordance with this, no colonies were formed on plates containing both antibiotics (Figure 2B), and we concluded that the 269 bp region cloned in pUTR269 contains an important incompatibility element. To determine whether this element could be an antisense RNA, a Northern blot was performed, which detected a small antisense RNA (<100 nt), both in PR01 (which carries pSA564) and in the strains harboring plasmid pUTR269 or pRacUTR (Figure 2C). These data are all consistent with an antisense RNA based plasmid replication control system of the same type as pSK41 and pSK1 (Kwong et al., 2006, 2008; Kwong and Firth, 2015), and we decided to name the antisense RNA from pSA564 “RNA1.”

### Modification of RNA1 Alters Plasmid Incompatibility Characteristics

**FIGURE 3:**
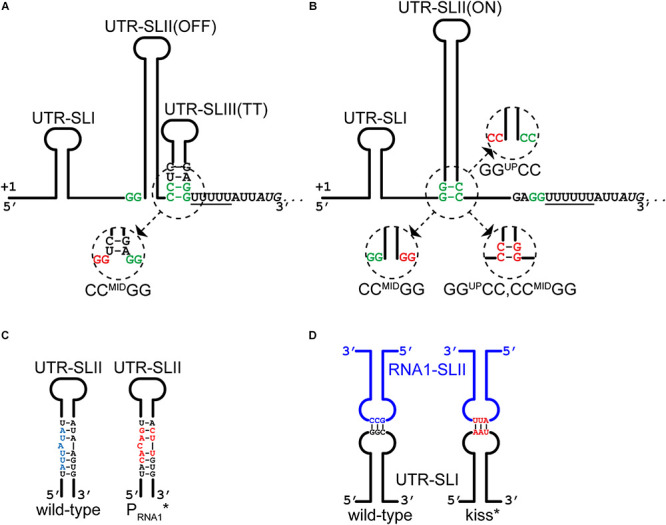
Mutations that alters RNA1-*repA* mRNA interactions. **(A)** The CC^MID^GG mutation (red nucleotides in insert) prevents the formation of the putative OFF-structure by shortening the duplex of the stem and liberating some of the nucleotides of the ribosome binding site. **(B)** The CC^MID^GG mutation (red nucleotides in insert) removes two base-pairs from the foot of the 46 base-pair UTR-SLII(ON) stem, in addition to preventing the putative OFF-structure from forming **(A)**. The GG^UP^CC mutation (red nucleotides in insert) also weakens the UTR-SLII stem, which presumably shifts the equilibrium toward formation of UTR-SLIII(TT). The GG^UP^CC,CC^MID^GG mutation (red nucleotides in insert) allows the full length of the UTR-SLII(ON) stem to form, and at the same time weakens/prevents the UTR-SLIII(TT). **(C)** The P_RNA__1_* mutations in the –10 region of the RNA1 promoter (red nucleotides) do not disrupt base-pairing within the UTR-SLII (wild-type in blue). See also Supplementary Figure 2 for full nucleotide sequence. **(D)** The kiss* mutations (in red) weaken the base-base interactions between RNA1-SLII and UTR-SLI. See also Supplementary Figure 2 for full nucleotide sequence.

The antisense RNA1 molecule potentially forms two stem loops (Zuker, 2003), RNA1-SLI and RNA1-SLII, where the downstream hairpin (SLII) doubles as a *rho*-independent transcriptional terminator due to the stretch of uridine residues that immediately follow it (Figure 1D). RNA1-SLII has a CGCCAA-loop, which could function as a “kissing loop” (Tomizawa, 1984; Forsdyke, 1995), i.e., the site of initial base-pairing between RNA1 and the *repA* mRNA 5′UTR (Figure 3D). RNA1 includes the dinucleotide CC^RNA1^, which is complementary to GG^UP^, and will sequester it upon forming a duplex with the mRNA 5′UTR, leaving CC^MID^ free for pairing with GG^DW^ (Figure 1E).

**FIGURE 4:**
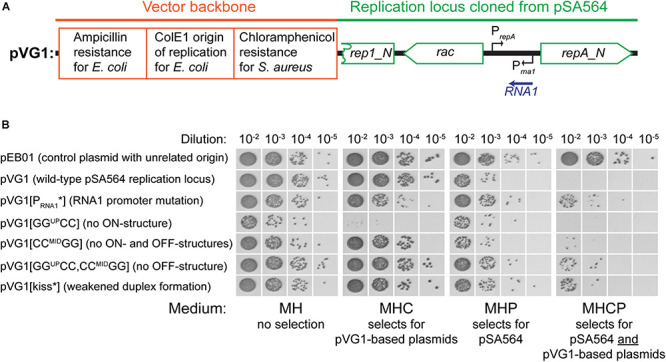
Incompatibility between pVG1 derivatives and pSA564. **(A)** Schematic linear map of the pVG1 construct. The orange section is from the vector backbone, which includes a ColE1 origin of replication for *E. coli* (but not for *S. aureus*), an ampicillin resistance cassette for *E. coli* and a chloramphenicol resistance cassette for *S. aureus*. The green section was cloned from pSA564, and includes (from left to right) a truncated *rep1_N* gene, the *rac* gene, the *RNA1* gene and the *repA_N* gene. The location of the *rac* promoter is unknown. Further details about the pSA564 insert can be found in Supplementary Figure 1. **(B)** The pVG1 plasmid and its derivatives were transformed into PR01, plated on MHC agar plates and incubated over night at 37°C. Colonies were picked directly from the transformation MHC agar plate and resuspended in MH medium. These suspensions were used for serial dilutions that were then spotted on MH, MHC, MHP, and MHCP agar-plates and incubated over night at 37°C. Growth on MHC indicates the presence of pVG1 or its derivatives (or the pEB01 control), growth on MHP indicates the presence of pSA564, and growth on MHCP indicates the presence of both pSA565 and a pVG1 plasmid. Details of the mutations in pVG1 can be found in Table 2.

To evaluate the importance of the individual sections of RNA1 and the *repA* mRNA 5′UTR, we generated pVG1, a plasmid that replicates in *S. aureus* via the pSA564 replication machinery and origin (Figure 4A). Based on data from pSK1 (Kwong et al., 2008), we expected the pSA564 replicon to be within the *rep1* to *repA_N* region (position 19005 to 21937) of pSA564, and this region was therefore cloned into plasmid pRLYC1, which carries a *S. aureus* chloramphenicol resistance gene and an origin of replication that is functional in *E. coli* but not in *S. aureus* (Supplementary Figure 1). pVG1 can therefore easily be cloned and modified in *E. coli*, but its *S. aureus* replication machinery and copy-number control are from pSA564.

**TABLE 2:** Overview of mutations in the *repA* 5′UTR.

**Name**	**Original nucleotides**	**Mutated to**	**Description**
GG^UP^CC	GG (pos. +46 and +47 on UTR)^*a*^	CC	Cannot form the putative ON structure
CC^MID^GG	CC (pos. +164 and +165 on UTR)^*a*^	GG	Can neither form the putative OFF-structure nor the putative ON-structure
GG^UP^CC, CC^MID^GG	GG and CC (pos. as above)^*a*^	CC and GG	Cannot form the putative OFF-structure
kiss*	GGC (pos. +28 to +30)^*a*^	AAU	Weakens the proposed loop-loop “kissing” interactions between RNA1 and the *repA* 5′UTR
P_RNA1_*	UUAUA (pos. +98 to +102) and UAA (pos. +117 to +119)	CACAG and CUU	−10 element of RNA1 promoter mutated from TATAAT to CTGTGT while retaining the base-pairing in the putative stem of UTR-SLII

*^*a*^These mutations also affect the sequence of the antisense RNA1.*

We then examined how RNA1 controls *repA* expression by modifying pVG1 in several ways: (i) abolishing the production of RNA1 (pVG1[P_RNA__1_^∗^]), (ii) weakening the putative kissing interaction between the RNA1-SLII loop and the *repA* mRNA (pVG1[kiss]), (iii) reducing formation of both putative secondary structures of the 5′UTR by mutating CC^MID^ to GG (pVG1[CC^MID^GG]), which at the same time will abolish duplex formation with the RBS, (iv) reducing formation of the proposed ON-structure of the 5′UTR by mutating GG^UP^ to CC (pVG1[GG^UP^CC]), or (v) reducing formation of the proposed OFF-structure of the 5′UTR by mutating both GG^UP^ to CC and CC^MID^ to GG (pVG1[GG^UP^CC, CC^MID^GG]) (summarized in Table 2 and Figure 3). These mutant plasmids were then introduced into PR01 where transformant colonies were selected by growth on MHC. Then, 10-fold dilution series of these colonies were spotted on MHC, MHP, and MHCP to detect the presence of the pVG1 variant, pSA564 and pSA564 AND pVG1 variant, respectively (Figure 4). MH medium was also included, as control for growth without requirement for a plasmid.

pVG1 was able to replicate in PR01 (confirming that position 19,005 to 21,937 of pSA564 does indeed contain the replication locus). Furthermore, only about 1 out of 50 PR01 cells retain pSA564 after transformation with pVG1 and over-night selection on MHC (compare panel MH with panel MHP) and the complete lack of colonies on MHCP shows that pVG1 is incompatible with pSA564. This incompatibility is somewhat diminished with pVG1[P_RNA__1_^∗^], pVG1[GG^UP^CC, CC^MID^GG and pVG1[kiss], where the combined pool of RepA from pSA564 and the pVG1 variant is apparently large enough to replicate both plasmids with simultaneous chloramphicol and penicillin selection (however, note the poor growth of these strains on MHCP, compared to the pEB01-carrying control strain). Cells transformed with pVG1[GG^UP^CC] grew poorly on MHC medium, consistent with a model where its 5′UTR is permanently in the putative OFF-structure, and pVG1[GG^UP^CC] replication is therefore dependent on *repA* expression from endogeneous pSA564 (Figure 4). Importantly, neither the unrelated vector pEB01 (which carries pT181 replication machinery), nor growth on chloramphenicol in itself caused loss of pSA564.

**FIGURE 5:**
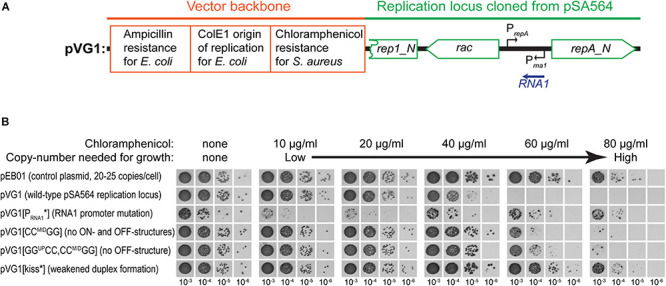
RNA1 mutations affect plasmid copy-number. **(A)** Schematic linear map of the pVG1 construct. The orange section is from the vector backbone, which includes a ColE1 origin of replication for *E. coli* (but not for *S. aureus*), an ampicillin resistance cassette for *E. coli* and a chloramphenicol resistance cassette for *S. aureus*. The green section was cloned from pSA564, and includes (from left to right) a truncated *rep1_N* gene, the *rac* gene, the *RNA1* gene, and the *repA_N* gene. The location of the *rac* promoter is unknown. Further details about the pSA564 insert can be found in Supplementary Figure 1. **(B)** The copy number of pVG1 and its mutant derivatives was estimated by plating on medium containing increasing concentrations of chloramphenicol. Growth at higher concentrations indicate higher chloramphenicol resistance gene dosage (i.e., higher plasmid copy number). The experiment was carried out in the PR02 (a RN4220 derivative) host strain, and plasmid replication is therefore whole dependent on *cis*-produced RepA. Details of the mutations in pVG1 can be found in Table 2.

To further analyze the role of RNA1 we also estimated the relative copy number of the plasmids by plating transformants on increasing concentrations of chloramphenicol. In the parental strain harboring pSA564, the resident plasmid could interfere with the copy number of pVG1, so the experiment was performed in PR02, a derivative of *S. aureus* RN4220 which harbors no plasmid. The wild-type version of the replicon, pVG1, was able to support growth up to 40 μg/ml chloramphenicol, whereas pVG1[GG^UP^CC, CC^MID^GG] and pVG1[CC^MID^GG] permitted growth at 60 μg/ml chloramphenicol, consistent with their inability to form the putative OFF-structure on *repA* mRNA (Figure 5). Strikingly, pVG1[P_RNA__1_^∗^] permitted PR02 to grow at a chloramphenicol concentration equal to that seen with cells harboring pEB01, which carries the pT181 replication genes, origin of replication and copy-number control mechanism (resulting in an expected copy number of 20 to 25 copies per cell) (Charpentier et al., 2004; Oun et al., 2013). Note that repeated attempts to transform PR02 with pVG1[GG^UP^CC] yielded no transformants, and this strain is therefore not included in the assay.

### RepA Protein Expression Is Regulated by RNA1 and the 5′UTR Structure

**FIGURE 6:**
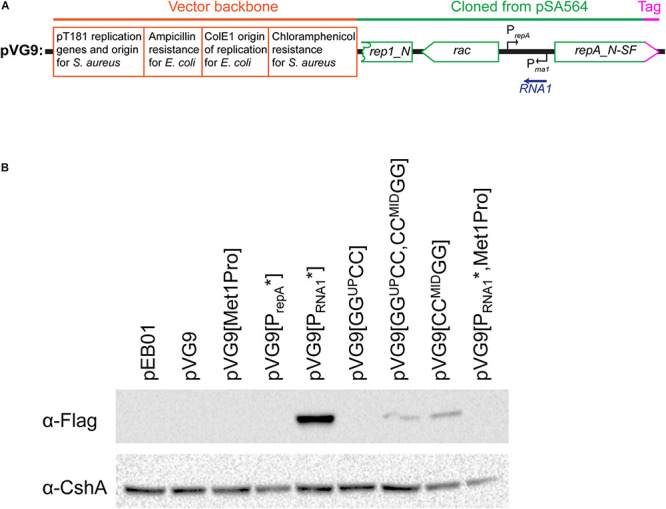
Mutating the RNA1 promoter permits the detection of RepA by Western blotting.**(A)** Schematic linear map of the pVG9 construct. The orange section is from the vector backbone, which includes a pT181 replication cassette (with replication genes and origin) for replication in *S. aureus*, a ColE1 origin of replication for *E. coli*, an ampicillin resistance cassette for *E. coli* and a chloramphenicol resistance cassette for *S. aureus*. The green section was cloned from pSA564, and includes (from left to right) a truncated *rep1_N* gene, the *rac* gene, the *RNA1* gene, and the *repA_N* gene. The latter is translationally fused to a streptavidine-FLAG tag (pink). The location of the *rac* promoter is unknown. Further details about the pSA564 insert and the backbone cloning vector can be found in Supplementary Figure 1. **(B)** RepA is undetectable under normal circumstances (pVG9) but becomes easily detectable when the RNA1 promoter is mutated (pVG9[P_RNA__1_^∗^]). An additional mutation of the RepA start codon abolishes detection (pVG9[P_RNA__1_^∗^, Met1Pro]), thus confirming the specificity of the antibody. Mutations that prevent formation of the putative OFF-structure (pVG9[GG^UP^CC,CC^MID^GG] and pVG9[CC^MID^GG]) also permits the detection of RepA. In the construct “P_*repA*_^∗^” the -10 box of the *repA* promoter was mutated from TAATAT to TAATGG, in “Met1Pro” the AUG start codon of RepA was mutated to CCG. In the pVG9 constructs the RepA was C-terminally fused to a streptavidine-FLAG tag, and was detected using anti-FLAG antibodies. Antibodies against the CshA protein were used as loading control.

The observed differences in chloramphenicol resistance in Figure 5 could hypothetically be due to altered expression of the chloramphenicol resistance gene (for example via changes to supercoiling density), but the base-pair differences between the various pVG1 constructs are so small (maximum 12 substitutions) that we consider this an unlikely explanation. Nevertheless, to circumvent this potential problem, we decided to directly measure the RepA levels, and confirm that they are impacted by the ON-OFF equilibrium. We constructed a plasmid (pVG9) where RepA-expression is still under control of the equilibrium between structures of the UTR and regulated by RNA1, but where the copy-number of pVG9 is defined by the pT181 replication genes and origin. In addition, the RepA protein in pVG9 was C-terminally fused with a Streptavidin-Flag-tag, in order to follow the expression of RepA by Western blotting with an anti-Flag antibody (Figure 6A and Supplementary Figure 1). A strong band was observed for pVG9[P_RNA__1_^∗^], which no longer produced RNA1 (Figure 6B). This band disappeared when the predicted *repA* start codon was mutated, not only confirming the identity of the detected protein but also experimentally verifying the annotation of the *repA* start codon (Figure 6B). It was also possible to detect a weak RepA expression from pVG1[GG^UP^CC, CC^MID^GG] and pVG1[CC^MID^GG] (Figure 6B), corresponding to the strains in which the equivalent pVG1 derivatives exhibited increased copy-number (Figure 5B).

### RNase J Plays a Major Role in the Control of Plasmid Replication

Mathematical modeling of negative regulators of plasmid replication which are constitutively expressed from the plasmid itself, predict that such regulatory molecules (e.g., RNA1) must have a short half-life (Pritchard et al., 1969). We therefore wanted to identify the host-factors needed to ensure rapid degradation of RNA1, and we argued that the lack of such a host-factor would lead to accumulation of RNA1 and subsequent loss of pSA564.

**FIGURE 7:**
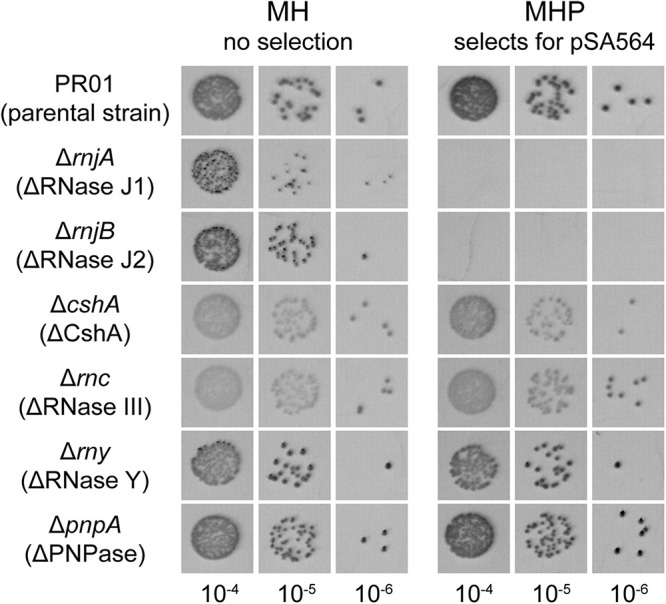
pSA564 is lost in RNase J1 and J2 mutants. SA564-derived strains, mutated for different components previously identified to be part of the *S. aureus* RNA degradation machinery, were spotted on MH and MHP. The presence of pSA564 was determined by growth on MHP. The strains mutated for the two genes, *rnjA* and *rnjB*, encoding the 5′–3′ ribo-exonucleases RNase J1 and RNase J2 were no longer able to form colonies on MHP plates.

We had previously obtained mutants of PR01 (which naturally carries pSA564, since PR01 is a derivative of SA564) where we had deleted *cshA*, *rny, pnpA, rnjA*, and *rnjB* (coding for CshA, RNase Y, PNPase, RNase J1 and RNase J2, respectively), and for this study, we additionnally generated a strain deleted for *rnc* (conding for RNase III). These mutants of the RNA decay machinery were spotted on penicillin plates to test for the presence of pSA564. All tested mutant strains were able to grow on penicillin, except Δ*rnjA* or Δ*rnjB* (Figure 7), leading us to suspect that RNase J1 and J2 were both required for degradation of RNA1 (and thereby for replication of pSA564).

RNase J1 and J2 mutants exhibit severe pleiotropic phenotypes (Linder et al., 2014). Therefore, to ensure that the penicillin sensitivity was not an artifact of these phenotypes, we re-analyzed our previously published whole genome sequencing data from our *rnjA* and *rnjB* mutants (Linder et al., 2014) and confirmed that no pSA564-derived sequences could be detected, which established that our RNase J1 and J2 mutants strains had indeed lost pSA564. However, these strains were mutants that had been generated several years ago (Redder and Linder, 2012), and we had no direct proof that their parental strain was carrying pSA564 when the mutations were made. We therefore decided to generate a new *rnjA* deletion mutant, this time in a parental strain where we had verified the presence of pSA564 immediately before deleting *rnjA*. Once again, we observed that pSA564 was lost upon the introduction of the *rnjA* mutation, proving that RNase J1 indeed is a key host-factor for this plasmid.

### RNA1 Is Overproduced in an *rnjA* Mutant

**FIGURE 8:**
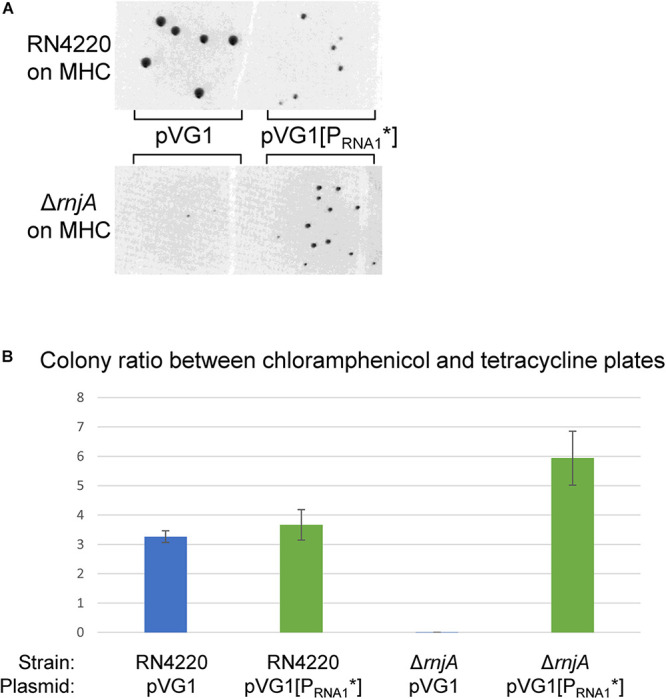
RNA1 is essential for RNase J-deficient plasmid-loss. **(A)** Both pVG1 and pVG1[P_RNA__1_*] are able to establish themselves in RN4220 (an *S. aureus* strain which does not harbor any plamids), although the colonies are visibly smaller for pVG1[P_RNA__1_*]. pVG1[P_RNA__1_*] is able to establish itself in the Δ*rnjA* strain to form colonies in 24 h. The RN4220 and the Δ*rnjA* colonies are from two different MHC-plates. **(B)** The colony counts from MHC-plates (pVG1 and pVG1[P_RNA__1_*] transformants) at ∼24 h were normalized with counts from MHT-plates (pCN36 transformants, counted at ∼42 h). RN4220 exhibits similar colony, colony ratios for pVG1 and pVG1[P_RNA__1_*] (blue and green bars, respectively), whereas only pVG1[P_RNA__1_*] replicates rapidly in Δ*rnjA* to form colonies within 24 h. Note that Δ*rnjA* transformed with pVG1 will eventually form colonies when the plates are left long enough in the incubator.

To verify that loss of pSA564 in the RNase J mutant strains was directly linked to RNA1, we used pVG1 and pVG1[P_RNA__1_^∗^] which contain only the replicon from pSA564. If pVG1[P_RNA__1_^∗^] replicates in the Δ*rnjA* strain, while pVG1 does not, then RNase J1 must influence replication via RNA1. To ensure that variations in transformation efficiencies would not lead to bias, we co-transformed the two plasmids with the un-related tetracycline resistance plasmid pCN36, in order to use the number of tetracycline resistant transformants as normalizer (Figure 8). The pVG1[P_RNA__1_^∗^] was readily established in the Δ*rnjA* strain, whereas the pVG1 plasmid was not, demonstrating that RNA1 is essential for the plasmid-loss observed in RNase J mutants. Performing the same experiments with RN4220 (a control strain which does not harbor any plamids) revealed no significant difference between pVG1 and pVG1[P_RNA__1_^∗^], except that colonies with pVG1[P_RNA__1_^∗^] were much smaller than colonies with pVG1 (Figure 8), presumably due to the added burden of un-controlled replication from the pSA564 origin.

**FIGURE 9:**
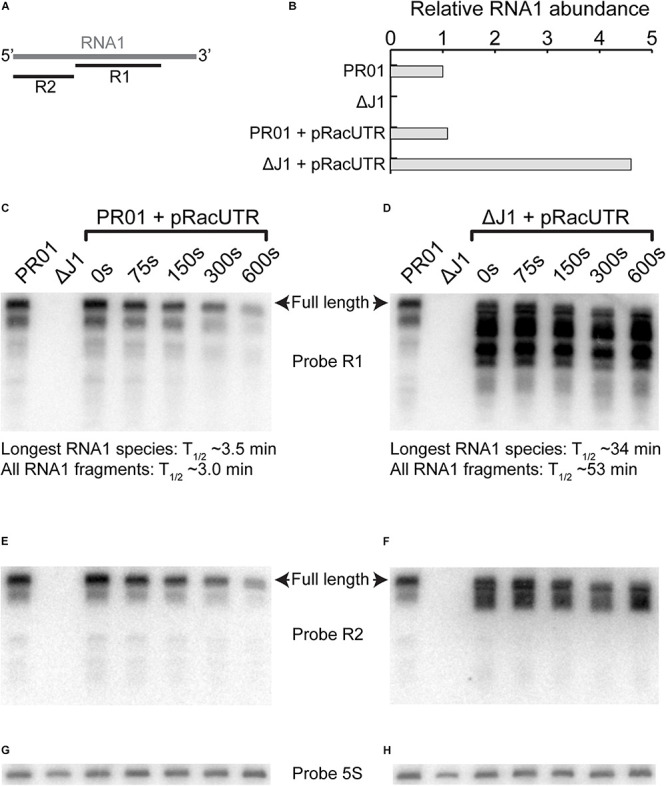
RNA1 degradation and abundance. **(A)** Overview of where probe R1 and R2 hybridizes on RNA1. **(B)** Level of RNA1 is similar for pSA564 and pRacUTR in PR01, but the overall intensity of the combined RNA1 bands are much higher in Δ*rnjA*. **(C,D)** Rifampicin was used to block transcription and RNA was extracted at different time points to follow the rate of degradation by Northern blotting. The intensity of longest RNA1 species detected for each time-point was used to calculate the RNA1 half-life. However, RNA1 fragments accumulate in the Δ*rnjA* mutant, and these shorter RNA1 fragments cannot be readily quantified individually, so we here present the half-life of the combined signal of the R1 probe (i.e., all bands pooled). **(E,F)** A second probe (R2) against RNA1, this time targeting the 5′-end was used to re-probe the same membrane as in **(C,D)**, respectively. **(G)** Probe against 5S rRNA to normalize the membrane shown in **(C,E)**. **(H)** Probe against 5S rRNA to normalize the membrane shown in **(D,F)**.

To determine whether the plasmid-loss effect was due to an accumulation of RNA1 (as would be expected in a mutant deficient in RNA degradation), we examined the stability of RNA1 in the wild-type and Δ*rnjA* strains (Figure 9). We were unable to do this directly in the Δ*rnjA* strain, since it has lost the pSA564 plasmid, so we transformed both the Δ*rnjA* and the parental PR01 strains with pRacUTR, which replicates via the pT181 origin of replication and expresses RNA1 and the *repA* 5′UTR (but not the *repA* coding region). Both RNAs are transcribed from their natural promoters and all potential RNA-RNA duplex-forming regions are intact, so we expected this to mimic the natural situation from pSA564. Indeed, the RNA1 levels in PR01 expressing RNA1 from pSA564 and from pRacUTR were similar (Figure 9B).

Northern blot analyses showed that the half-life of the longest RNA1 species increase 10-fold in the RNase J1 mutant (Figures 9C,D, compare the upper band in the two “0 s” lanes). To our slight surprise the level of longest RNA1 species remains similar in the wild-type and the Δ*rnjA* strains. However, several RNA1 fragments were observed to strongly accumulate in the Δ*rnjA* strain, due to a 17-fold average increase in their half-lives (Figure 9D).

### The 3′-Section of RNA1 Is Sufficient for Replication Inhibition

We hypothesized that the 3′-section of RNA1 which accumulates in Δ*rnjA* should be able to inhibit pSA564 replication. However, can such short RNA molecules still compete with the intra-molecular secondary structures of the *repA* UTR to form an RNA1-UTR duplex? And would such a duplex really prevent formation of the proposed ON-structure?

Inspection of the RNA1-UTR duplex and the putative secondary structures of the UTR suggested that RNA1 molecules truncated (or degraded) at their 3′ ends are likely to retain their ability to compete with the inferred UTR-SLII(ON) structure. However, RNA1 molecules that are truncated at their 5′ ends will overlap less with this UTR-SLII(ON) structure, and might therefore be less efficient in shifting the UTR to the OFF-structure. To determine the lower limit for a “competitive” RNA1 fragment, we constructed two versions of pRacUTR which express RNA1 molecules shortened at their 5′ ends by 18 nt (pRNA1-18nt) and 38 nt (pRNA1-38nt), respectively. It should be noted that both these truncated RNA1s carry the GG dinucleotide which is antisense to the key CC^MID^ nucleotides of the UTR. pRNA1-18nt was incompatible with pSA564 (Supplementary Figure 3C). However, the shorter RNA1-38nt was unable to cause the loss of pSA564, and Northern blotting therefore detected both full-length RNA1 from pSA564 and RNA1-38nt from pRNA1-38nt (Supplementary Figure 3D).

## Discussion

Before this study, it was already known that the two RepA_N-family plasmids pSK1 and pSK41 produce a small antisense RNA as copy-number control molecule from the opposite strand of their *repA_N* 5′UTR. Our highly similar findings for pSA564 show that this asRNA could be a universal system for this plasmid family. Indeed, a survey of small RNAs from *S. aureus* N315, using next-generation sequencing, detected an uncharacterized antisense RNA in the 5′UTR region of the predicted *repA_N* gene of pN315 (Beaume et al., 2010), further strengthening this hypothesis.

### Genetic Analyses of the pSA564 Replication Control Mechanism

Both pUTR269, which only carries a 269 bp region of pSA564, as well as pVG1, which replicates in *S. aureus* via a pSA564 minimal replicon, are incompatible with the extant pSA564 (Figures 2, 4). Moreover, mutations in the promoter region of RNA1 in pVG1 strongly increased compatibility with the endogenous pSA564 (Figure 4), confirming that RNA1 is an important incompatibility element.

An examination of the relative copy-number of the pVG1 variants (Figure 5) suggested that the lack of RNA1 (in pVG1[P_RNA__1_^∗^]) increases the copy-number, as would be expected when an inbitor of RepA expression is removed. However, the bacteria grow poorly when carrying the plasmid without copy-number control (see colony size with pVG1[P_RNA__1_^∗^] in Figure 5B and compare left and right upper panel in Figure 8A), and we speculate that the uncontrolled plasmid replication results in a significant added burden to the cell. Interestingly, the mutation in the putative kissing loop of RNA1-SLII and UTR-SLI (pVG1[kiss^∗^]) raised the copy-number to the same level as pVG1[P_RNA__1_^∗^] (Figure 5B), demonstrating that these three nucleotides play a key role in the regulatory mechanism. pVG1[GG^UP^CC, CC^MID^GG] and pVG1[CC^MID^GG] exhibit an increased copy-number compared to pVG1 (Figure 5B), but their effect is smaller than for pVG1[P_RNA__1_^∗^] and pVG1[kiss^∗^], suggesting that the CC^MID^GG mutation does not completely eliminate negative regulation of RepA expression. pVG1[GG^UP^CC] prevents the ON-structure from sequestering the CC^MID^ dinucleotide, and thus allows the full-length putative UTR-SLIII(TT) to form irrespective of RNA1. Our inability to transform PR02 with pVG1[GG^UP^CC], therefore suggests that RepA expression is completely abrogated when the stem of UTR-SLIII(TT) includes base-pairing between CC^MID^ and GG^DW^ (Figure 1B) (note that in contrast to PR01, PR02 cannot provide RepA *in trans*, since it does not carry pSA564; see Table 3).

**TABLE 3:** Strains.

**Short name**	**Strain name**	**Chromosomal genotype**	**Plasmid content**	**References**
SA564	*S. aureus* SA564	Parental strain	pSA564	Giraud et al., 2015; Accession number: CP010890.1
RN4220	*S. aureus* RN4220	Parental strain	None	Nair et al., 2011
PR01	PR01	SA564 *hsdR* type III mutant Δ*pyrFE*	pSA564	Redder and Linder, 2012
PR02	PR02	RN4220 Δ*pyrFE*	None	Redder and Linder, 2012
ΔRNase J1	PR01-01	PR01; Δ*rnjA*	None	Redder and Linder, 2012
ΔRNase J2	PR01-04	PR01; Δ*rnjB*	None	Redder and Linder, 2012
ΔRNase Y	PR01-02	PR01; Δ*rny*	pSA564	Redder and Linder, 2012
ΔRNase III	SVK97.1	PR01; Δ*rnc*	pSA564	This study
ΔPNPase	PR01-07	PR01; Δ*pnpA*	pSA564	Redder and Linder, 2012
ΔCshA	PR01-15	PR01; Δ*cshA:kanA*	pSA564	Linder et al., 2014
ΔRNase J1new	VG_J1new	PR01; Δ*rnjA*	none	This study

The levels of RepA expressed from the various pVG9 mutants (Figure 6) correspond to the observations of relative plasmid copy number (Figure 5). The RepA control mechanism is designed to shut off expression when the copy number rises, and the medium-copy number of the pVG9 constructs (determined by the pT181 replication mechanism and origin) was therefore expected to lead to a firm repression of RepA expression. Furthermore, natural levels of RepA are presumed to be very low, since RepA from pSK41 was undetectable (Kwong et al., 2006; Liu et al., 2012). We were indeed unable to detect the RepA protein from pVG9, presumably because the gene dosage of RNA1 goes up with increased copy-number. However, RepA becomes easily detectable when the RNA1 promoter is mutated (in pVG9[P_RNA__1_^∗^]), confirming that RNA1 controls the level of RepA, which is necessary for the initiation of plasmid replication.

Taken together, the data from the pVG1 and pVG9 mutants of the *repA_N* 5′UTR constitute compelling genetic evidence for a plasmid replication control model where RNA structures that are similar (or identical) to those proposed in Figure 1 are in an equilibrium, which is shifted according to the intracellular concentration of RNA1.

### RNase J1 Degradation of RNA1 Is Essential for pSA564 Replication

Of the RNA decay mutants tested in this study, only RNase J1 and J2 mutants had lost pSA564 (Figure 7). RNase J1 and J2 have previously been proposed as essential for degrading the toxin mRNA in the *par* toxin-antitoxin system of pAD1 from *Enterococcus faecalis* (Shokeen et al., 2009), and we cannot exclude that pSA564 carries a similar *par*-like toxin-antitoxin system which killed all plasmid-carrying cells when RNase J1 or J2 was deleted (leaving only the cells that have lost pSA564 to form colonies). However, pVG1 only contains a short section of pSA564, without room for any putative toxin-antitoxin systems, and the lack of pVG1 replication in our RNase J1 mutant (Figure 8) therefore excludes that RNase J1 is needed for inhibiting toxin activity. Moreover, the lack of RNA1 is epistatic to the lack of RNase J1, since pVG1[P_RNA__1_^∗^] was able to replicate in the RNase J1 mutant (Figure 8). From this we conclude that RNase J1 (and presumably also RNase J2) promotes pSA564 replication via degradation of RNA1.

We examined RNA1 stability in the natural host of pSA564, and as expected it was quite short (about 3.5 min, Figure 9C), but we were able to detect a large half-life increase and concomittant accumulation of RNA1 fragments in the RNase J1 mutant (Figures 9D,F). We propose that these accumulating RNA1 fragments are responsible for blocking RepA expression (and consequent plasmid loss), and we show that RNA1 fragments with 18 nts missing from the 5′ end (RNA1-18nt) can indeed perform this role (Supplementary Figure 3). However, when 38 nts are removed (and this includes the putative RNA1-SLI hairpin), then the inhibitory effect is abolished (Supplementary Figure 3), which suggests that the putative RNA1-SLI secondary structure is required for RNA1 to shift the equilibrium of the *repA* 5′UTR toward a structure that blocks RepA expression.

Our Northern blot revealed a slight decrease in the size of RNA1 after 300 and 600 s in the wild-type as well as in the Δ*rnjA* strain (Figures 9C,D), which is presumably due to “nibbling” by 3′ exoribonucleases. This hypothesis is further strengthened by the observation that some RNA1 fragments that accumulate in the Δ*rnjA* mutant have been shortened at their 3′ ends (and are therefore still visible with the R2 probe) while others have lost their 5′ ends (visible with the R1 probe and undetected with the R2 probe) (Figures 9E,F).

Interestingly, the long RNA1 fragments that are visible with both the R1 and R2 probes in the Δ*rnjA* RNA are not detected in the wild-type strain (Figures 9E,F), and it seems likely that these are normally degraded from their 5′ end by the 5′ to 3′ exoribonucleolytic activity of RNase J1. In contrast, the fragments that have lost their 5′ ends in the Δ*rnjA* RNA (undetected with the R2 probe, compare Figures 9D,F), must have been generated by an unidentified endoribonuclease, and only then degraded by RNase J1. It therefore appears that RNase J1 is not always the initator of RNA1 degradation, but that RNase J1 is required for degrading the 3′ region of RNA1, i.e., a region which is sufficient for controlling pSA564 replication (see RNA1-18nt in Supplementary Figure 3).

RNases have previously been observed to be required for plasmid replication in Gram-negative bacteria via degradation of regulatory RNAs (Saramago et al., 2015 and references therein), but this has to our knowledge not previously been seen in Firmicutes. In *E. coli*, for example, it is RNase E which initiates degradation of the asRNAs RNAI and CopA, which negatively regulate replication of the plasmids ColE1 and R1, respectively (Lin-Chao and Cohen, 1991; Söderbom et al., 1997). The downstream RNase E cleavage products of RNAI and CopA are also able to inhibit plasmid replication (similar to RNA1-18nt, discussed above), and the rapid degradation of these intermediates requires the PAP I poly(A)polymerase. Mutating PAP I lowers the copy-numbers of both ColE1 and R1, but does not cure these plasmids (Xu et al., 1993; Söderbom et al., 1997). RNase E is unfortunately essential in *E. coli*, but it is possible that deleting RNase E would have the same dramatic effect that we observe for pSA564 when we delete RNase J1. Interestingly, *S. aureus*, *B. subtilis*, and many other Firmicutes encode neither RNase E nor PAP I homologs (Campos-Guillén et al., 2005; Durand et al., 2015), and it is possible that RNAI and CopA would be too stable to allow ColE1 and R1 replication in Firmicutes. Hfq, a protein which normally promotes RNA-RNA duplex formation in *E. coli*, appears to directly or indirectly prevent RNAI from interacting with the replication pre-primer RNAII, thus increasing replication of ColE1-like plasmids (Cech et al., 2014). While *S. aureus* does encode an Hfq homolog, it does not appear to be implicated in RNA-RNA interactions, suggesting a different organization of sRNA-mediated regulation (Bohn et al., 2007).

### Types and Activities of Host-Encoded RNases Could Be a Major Factor in Limiting Plasmid Host-Ranges

We show here that RNase J1 and J2 are essential host-factors for pSA564 replication. Their activity will therefore be a key determinant for the host-range of pSA564. An NCBI database search (February 2019) revealed 94 plasmids with a *repA* UTR region with 100% identity to the pSA564 UTR and 22 plasmids in which the whole *repA* locus is identical to that of pSA564. Notable amongst these is pSA564-fus which was recently isolated from a Danish patient and is identical to pSA564 except for the additional presence of a *fusB* fusidic acid resistance cassette (Edslev et al., 2018). The dependence on RNase J probably extends to all plasmids with pSA564-like replication origins, as well as to other members of the *S. aureus* RepA_N plasmid family, since all appear to be regulated by RNA1-UTR-type mechanisms and have narrow host-ranges (Weaver et al., 2009).

RNase J homologs are found in several bacterial phyla, but are conspicuously absent from Bacteroidetes, Chlamydiae, beta-proteobacteria and the majority of gamma-proteobacteria (for example *E. coli*) (Laalami et al., 2014). Moreover, the Firmicutes generally encode at least two RNase J paralogs (for example RNase J1 and J2 in *S. aureus*). Each paralog has different activities and specificities, which are modified when the paralogs form a hetero-protein complex (Mathy et al., 2010; Linder et al., 2014; Raj et al., 2020). There is also variation in the relative importance of different RNases, depending on the species. For example, deletion of RNase J1 or J2 in *S. aureus* causes severe growth defects and leads to the accumulation of hundreds of RNA species (Linder et al., 2014), whereas in *B. subtilis* it is RNase Y deletion mutants that exhibit the most severe defects (Figaro et al., 2013). In addition, the 5′ exoribonuclease activity of RNase J homologs prefer substrates that are 5′ mono-phosphorylated, and each bacterial species encodes a different set of RNA pyrophosphohydrolases, which are enzymes that convert tri-phosphates into mono-phosphates at the 5′ ends of RNA (i.e., tri-phosphorylation of the +1 ribonucleotide into mono-phosphorylation) (Mathy et al., 2010; Hausmann et al., 2017; Srouji et al., 2017).

These species-dependent variations between RNase J-proteins and the RNA pyrophosphohydrolases would modify the speed of RNA1 decay as well as the type of RNA1 fragments that accumulate, meaning that the copy-number of an RepA_N plasmid would be different in each potential host species. If the endogenous RNase J enzymes are not able to degrade RNA1 correctly, then the RepA_N plasmid would not replicate and if RNA1 is degraded too readily, then the RepA_N plasmid would replicate too fast and put a burden on the host cell (we observe this when the pVG1[P_RNA__1_^∗^] construct leads to slow-growing colonies in Figures 5B, 8A). Such slower growing bacteria will presumably be outcompeted in nature, unless a continuous selection pressure is maintained (for example with penicillin).

As discussed above, it is expected that negative regulators of plasmid replication are rapidly degraded to ensure that plasmids can coordinate their replication with cell volume. RNAIII from pIP501 is an example of a very stable replication-inhibiting asRNA, with a half-life of about 30 min in *B. subtilis* (Brantl and Wagner, 1996). This should preclude proper copy-number control, and the authors propose an elaborate model where the plasmid-encoded transcriptional regulator CopR represses both RNAIII and its target *repR* mRNA in order to control replication (assuming that CopR has a short half-life; Brantl, 2015, and references therein). Intriguingly, pIP501 was discovered in *Streptococcus agalactiae* and presumably has a copy-number control mechanism which has evolved to fit the RNA decay system in that bacterium. Little is known about RNA decay in *S. agalactiae*, but both RNase J1 and J2 are essential in *Streptococcus pyogenes*, whereas they are both non-essential in *B. subtilis* (Bugrysheva and Scott, 2010; Figaro et al., 2013). It is therefore possible that while RNAIII exhibits a long half-life in *B. subtilis* (Brantl and Wagner, 1996), it may have a short half-life (leading to more precise copy-number control) in *S. agalactiae* where the activity and specificity of the RNA degradation system might be different than in *B. subtilis*.

The host-range of the RepA_N family plasmids is of clinical relevance, since they readily pick up antibiotic resistance cassettes and virulence factors from the chromosome or other plasmids (Weaver et al., 2009; Liu et al., 2013), and their horizontal dispersal therefore directly impacts global health. The importance of an RNase as essential host-factor is highlighted by the fact that the copy-number of many other plasmid families (e.g., ColEI, R1 and pT181) is also regulated negatively by short RNA molecules (del Solar and Espinosa, 2000). The paradigm for such regulation is that the regulating molecule must be short-lived (Pritchard et al., 1969; Wagner and Brantl, 1998), and it is therefore probable that RNase activities and specificities play a large role in determining the host-range of plasmids in general.

## Materials and Methods

### *Staphylococcus aureus* Strains and Growth Conditions

Strains and plasmids used in this study are described in Tables 3, 4, respectively.

**TABLE 4:** Plasmids.

**Plasmid name**	**Parent**	**Comment**	**References**
pSA564	N/A	Penicillin resistance. Accession number: CP010891.1	Giraud et al., 2015
pCN47	N/A	Erythromycin resistance	Charpentier et al., 2004
pCN36	N/A	Tetracyclin resistance	Charpentier et al., 2004
pEB01	pCN47	Chloramphenicol resistance; pT181 origin for *S. aureus* and ColE1 origin for *E. coli*	Oun et al., 2013
pSauJ1^Δ^ ^2–565^	pEB01	SA0941 and its promoter with the start codon of the following gene fused directly to a streptavidin-flag tag	Hausmann et al., 2017
pRLYC1	N/A	Chloramphenicol resistance; no replication origin for *S. aureus*, ColE1 origin for *E. coli*	Redder and Linder, 2012
pUTR269	pEB01	Nucleotides 20527-20795 from pSA564 cloned into *Sal*I and *Bam*HI	This study
pRacUTR	pEB01	Nucleotides 18580-20795 from pSA564 cloned into *Sal*I and *Bam*HI	This study
pVG1	pRLYC1	Nucleotides 19005-21937 from pSA564 cloned into *Sal*I and *Bam*HI	This study
pVG1[GG^UP^CC]	pVG1	GG to CC (pos. +46 and +47 on UTR)	This study
pVG1[CC^MID^GG]	pVG1	CC to GG (pos. +164 and +165 on UTR)	This study
pVG1[GG^UP^CC,CC^MID^GG]	pVG1	GG to CC (pos. +46 and +47 on UTR) and CC to GG (pos. +164 and +165 on UTR)	This study
pVG1[kiss*]	pVG1	GGC to AAT (pos. +28 to +30 on UTR)	This study
pVG1[P_RNA1_*]	pVG1	TTATA to CACAG (pos. +98 to +102 on UTR) and TAA to CTT (pos. +117 to +119 on UTR)	This study
pVG9	pSauJ1^Δ^ ^2–565^	Nucleotides 19005-21734 from pSA564 inserted between *Sal*I and *Age*I, which removes the SA0941 and its promoter, but leaves the streptavidin-flag tag fused to the C-terminal of RepA	This study
pVG9[GG^UP^CC]	pVG9	GG to CC (pos. +46 and +47 on UTR)	This study
pVG9[CC^MID^GG]	pVG9	CC to GG (pos. +164 and +165 on UTR)	This study
pVG9[GG^UP^CC,CC^MID^GG]	pVG9	GG to CC (pos. +46 and +47 on UTR) and CC to GG (pos. +164 and +165 on UTR)	This study
pVG9[kiss*]	pVG9	GGC to AAT (pos. +28 to +30 on UTR)	This study
pVG9[P_RNA1_*]	pVG9	TTATA to CACAG (pos. +98 to +102 on UTR) and TAA to CTT (pos. +117 to +119 on UTR)	This study
pVG9[Met1Pro]	pVG9	AUG to CCG (pos. +198 to +200 on UTR)	This study
pVG9[P_repA_*]	pVG9	TAATAT to TAATGG (pos. 20584-20589 on pSA564)	This study
pVG9[P_repA_*,Met1Pro]	pVG9	TAATAT to TAATGG (pos. 20584-20589 on pSA564) and AUG to CCG (pos. +198 to +200 on UTR)	This study

The *Escherichia coli* DH5α strain was grown in LB medium supplemented, if necessary, with 100 mg/l ampicillin (Sigma-Aldrich, Buchs, Switzerland). The *S. aureus* strains were cultivated in Mueller-Hinton broth supplemented with 20 mg/l uracil (MH) and when needed with 10 mg/l erythromycin (MHE), 10 mg/l chloramphenicol (MHC), 2 mg/l tetracyclin (MHT), 240 μg/l of penicillin G (MHP) (Sigma-Aldrich, Buchs, Switzerland), or a combination of chloramphenicol and penicillin G (MHCP). Agar plates contained 13 g/l of agar (Agar bacteriology grade, PanReac AppliChem). Strain PR01 is a derivative of SA564 where the restriction systems have been inactivated to facilitate transformation, and the *pyrFE* genes have been deleted to facilitate genetic manipulations, but where pSA564 replication remains unchanged (Corvaglia et al., 2010; Redder and Linder, 2012). Strain PR02 is a derivative of *S. aureus* RN4220, where the *pyrFE* genes have been deleted (Redder and Linder, 2012). Mutants SVK97.1 and VG_J1new were generated from PR01 using the protocol described previously (Redder and Linder, 2012).

### Molecular Biology Methods

All methods of standard molecular biology techniques used, were performed by the methods of Molecular cloning: a laboratory manual (Sambrook and Russel, 2001) or according to the recommendations of the manufacturers. Restriction enzymes were from New England Biolabs (Ipswich, MA, United States) and PCR products used for cloning were amplified using Q5^®^ High-Fidelity DNA Polymerase (New England Biolabs).

Primers used for vector construction are shown in Supplementary Table 1 in the Supplementary Material.

Sequencing of plasmids for verification was done at Fasteris SA (Plan-les-Ouates, Switzerland) and full genome sequencing was performed by the iGE3 genomics platform of Geneva University.

### Transformation Experiment

1 μg of the chloramphenicol resistance plasmids pVG1 and pVG1[P_RNA__1_^∗^] were each mixed with 250 ng pCN36 control plasmid, which carries a tetracyclin resistance cassette. The two plasmid mixtures were transformed into RN4220 and PR01-01 strains and plated on MHC and MHT plates. pVG1 and pVG1[P_RNA__1_^∗^] transformation is scored as the number of colonies growing on MHC divided by the number of colonies growing on MHT. RN4220 was chosen as control strain, since the parental strain of the PR01-01 strain carries pSA564 (which would compete with the pVG1 plasmids). The colonies were counted after 24 h incubation, except for PR01-01 on MHT, where it takes 40 h to grow visible colonies.

### Spotting Dilutions to Determine Growth Defects

All the different strains were cultivated overnight in MH liquid medium supplemented with adequate antibiotics if needed. Dilution series were then made in MH medium, to obtain 10^–2^ to 10^–6^ dilutions, 7.5 μl of which were spotted on MH medium and on MH supplemented with the appropriate antibiotic. Subsequently, the plates were incubated at 37°C until the WT colonies reached an appropriate size for photography. All the strains compared in the figures were spotted on the same agar plate.

### RNA Isolation

For RNA isolation, cultures were grown until mid-exponential phase (OD_600_ of ∼0.4) and harvested by rapidly mixing with five volumes of cold ethanol/acetone (1:1 vol:vol). Cells were pelleted (4,000 g for 5 min), washed in 1 ml TE buffer, resuspended in 200 μl TE with 10 μg Lysostaphin (Sigma) and 40 U RNasin^®^ Plus (Promega), and incubated at 37°C for 10 min. The lysed cells were mixed with 750 μl of TRIzol^TM^ Reagent (INVITROGEN) and 150 μl of chloroform. The samples were shaken vigorously for 15 s and left at room temperature for 3 min. Phase Lock Gel tubes (5prime, Hilden, Germany) were used to separate the phases, according to the manufacturer’s protocol. Approximately 300 μl of the solution from the aqueous phase were then transferred into a new 1.5 mL microcentrifuge tube. 20 μg of RNase-free glycogen was added, together with 375 μl cold isopropanol. The samples were then vortexed and stored overnight at −20°C. The RNA was pelleted by 40 min of centrifugation at 12,000 × *g* and 4°C. The supernatant was discarded, whereupon the pellet was washed with 1 mL of 75% cold ethanol and then centrifuged for 10 min at 7,500 × *g* at 4°C. The supernatant was discarded; the pellet was air dried for 15–20 min and the RNA pellet was resuspended in 20 μL of TE 1× buffer.

### RNA Half-Life Determination

Overnight cultures were diluted 1:100 into fresh media and grown to OD_600_ of ∼0.4. 400 μg/ml rifampicin was added to the cultures and samples were taken at five time points: 0, 75, 150, 300, and 600 s. Each sample of 6 ml was harvested by rapid mixing with 30 ml cold 1:1 ethanol:acetone solution.

### Northern Blot

Northern Blots were performed as in Khemici et al. (2015) with 8% acrylamide gels containing 8 M urea. 4 to 8 μg of total RNA (depending on experiment) was loaded in each lane and the marker was RiboRuler Low Range RNA Ladder (Thermo Scientific). The RNA migration was performed for 2 h at 90V. After electrophoresis, the RNA was transferred to a Hybond-N + membrane (GE Healthcare) for 2h at 30V and 0°C, whereupon the RNA was UV crosslinked to the membrane (120 kJ/cm^2^ Joules with a Stratalinker 2,400, Stratagene). Methylene blue staining was used to visualize the marker. The DNA oligo probes were 5′ labeled with ATP [γ-^32^P] and Polynucleotide Kinase (Thermo Fischer Scientific) and hybridized to the membrane over night at 37°C in ExpressHyb hybridization solution (Clontech, Mountain View, CA, United States). The signals were detected using a Typhoon FLA 7000 phosphorimager (General Electric). The membranes were stripped for 2 h with stripping solution (0.2% SDS and 10 mM Tris pH 7.5) before each new hybridization, and the stripping was verified by phosphoimager. Probe R1: TTGGCGTAGCATCGACTCTCGGTAATAAAACGATTCGCA, Probe R2: ATTCGTCTGTTTATATAATTTTTTG, 5S rRNA probe: TTAACTTCTGTGTTCGGCATGGGAACAGGTGTGA CCTCC.

### Western Blot

Bacterial cultures at OD_600_ of ∼0.4 (20 mL) were centrifuged (5,000 × *g*, 4°C, 10 min) and the pellet was lysed with 1 mL of PBS buffer (MgCl2 + CaCl2) (DPBS D8662, Sigma-Aldrich), anti-protease (cOmplete, Mini, EDTAfree Protease Inhibitor Cocktail, Sigma-Aldrich), lysostaphin (Ambi products, Lawrence, NY, United States) to a final concentration of 200 μg/ml and DNase I (M0303S; New England Biolabs) at a final concentration of 1 μl/ml for 15 min at 37°C. Protein levels were quantified by Bradford solution diluted five times (Bio-Rad Assay Dye Reagent Concentrate, #500-0006). Equal amounts (10 to 20 μg total protein) of lysates were prepared in loading buffer (4x = bromophenol blue 0,4% + 200mM Tris pH 6,8 + 40% glycerol + 8% SDS), migrated on precast 8% SDS-PAGE Gel (Eurogentec, ID-PA4121-015) in 1× MOPS buffer at 150V for 1 h. The PVDF membrane was activated by 30 s in methanol, 5 min in water and 10 min in 1× transfer buffer (tris base 0.25 M and glycine 1.9 M). After electrophoresis, proteins were transferred to PVDF membranes in 1× transfer buffer and 20% ethanol for 2 h at 100V at 4°C. Protein A was blocked by incubation over night at 4°C in 1× TBS, 5% milk, 0.1% Tween 20 and 1/500 human serum. The membranes were then incubated with a primary anti-FLAG antibody (Sigma-Aldrich, #F3165) or anti-CshA antibody and a secondary antibody Goat anti-mouse fused to HRP (Bio-Rad, #1706516). RepA-FLAG was revealed using Chemiluminescent Western Blot Detection kit (Thermo Fisher Scientific, #RPN2232) and the signals were detected using Bio-Rad ChemiDoc MP Imager.

## Data Availability Statement

The original contributions presented in the study are included in the article/Supplementary Material, further inquiries can be directed to the corresponding author/s.

## Author Contributions

VG, AL, VK, SH, AJ, CM, and PR performed the experiments. VG, JA, JP, PL, and PR analyzed the data. VG, AJ, PL, and PR wrote the manuscript. PL and PR conceptualized the work. All authors contributed to the article and approved the submitted version.

## Conflict of Interest

The authors declare that the research was conducted in the absence of any commercial or financial relationships that could be construed as a potential conflict of interest.
