# Supplementary material for: RNase J1 and J2 Are Host-Encoded Factors for Plasmid Replication
Source: Front Microbiol. 2021 May 4;12:586886. doi: 10.3389/fmicb.2021.586886 (PMC8129170; doi:10.3389/fmicb.2021.586886)

RNase J1 and J2 are host-encoded factors for plasmid replication

Vanessa Andrade Guimaraes[1], Alexandre Le Scornet[2], Vanessa Khemici[1], Stéphane Hausmann[1], Joshua Armitano[1], Julien Prados[1], Ambre Jousselin[2], Caroline Manzano[1], Patrick Linder[1], Peter Redder[1,2,*]

1) Department of Microbiology and Molecular Medicine,
Faculty of Medicine, University of Geneva
1, Rue Michel Servet, CH-1211 Geneva 4, Switzerland
2) Laboratoire de Microbiologie et Génétique Moléculaires,
Centre de Biologie Integrative, Paul Sabatier University
118, Route de Narbonne, 31062 Toulouse, France

*) Corresponding author: peter.redder@univ-tlse3.fr

Key words: *Staphylococcus aureus*; plasmid replication control; RNase J; antisense RNA; essential host factors

SUPPLEMENTARY DATA:

Table S1, Oligos:

Oligo usage	Name	Sequence
pRacUTR	repA-UTR-Sal-F4	TATTATGTCGACTATCAGTTAAAAAGTCAGATG
pRacUTR	repA-UTR-Sal-F4	TATTATGTCGACTATCAGTTAAAAAGTCAGATG
pUTR269	repA-UTR-Sal-F1	TAATAAGTCGACAGTTACAATGTTCTTTCAACTAA
pUTR269	repA-UTR-Bam-R1	TAATAAGGATCCCATAATAAAAACCTCATTTCCAC
pVG1	repA-Cop-Bam-F3	TATTATGGATCCTTACCATCTTCGGCATCGTC
pVG1	repA-UTR-Sal-F4	TATTATGTCGACTATCAGTTAAAAAGTCAGATG
pVG9	repA-UTR-Sal-F4	TATTATGTCGACTATCAGTTAAAAAGTCAGATG
pVG9	OVG-R-46	TACTCTACCGGTTTGATTAAAGATATTATATTG
P_{RNA1}*	OVG-R-28	TTGTACTTGCACAAGTATGATTAGATATACTGTGTACAAGTATCAAAAAAT TATA
P_{RNA1}*	OVG-F-29	CACAGTATATCTAATCATACTTGTGCAAGTACAAAACATAT
$GG^{UP}CC$	OVG-F-49	ATCGACTCTCCCTAATAAAACGATTCGCATTC
$GG^{UP}CC$	OVG-R-65	GTTTTATTAGGGAGAGTCGATGCTACGCCAAT
$CC^{MID}GG$	OVG-F-53	ACGTAAAAATTGTTTTATTAGGTCTATCCAAAAAGTGGAAATGAGG
$CC^{MID}GG$	OVG-R-82	CCTCATTTCCACTTTTTGGATAGACCTAATAAAACAATTTTTACGTAGGT
kiss*	OVG-R-55	ACCGAGAGTCGATGCTACTATTATAGTATCGACTCTTTTA
kiss*	OVG-R-56	AGTCGATACTATTAATGTAGCATCGACTCTCGGTAATAAAAC
Met1Pro	OVG-R-81	TTTCTCAAAATTGGGCCCAATAAAAACCTCATTTCCACTTT
Met1Pro	OVG-F-51	GGTTTTTATTCCGCCCAATTTTGAGAAATAC
P_{RepA}*	OVG-R-58	ATATTCACAAAGTACCATTAACACTTTTAAGATTGTATATAAAGCTT
P_{RepA}*	OVG-F-57	ATCTTAAAAGTGTTAATCCTACTTTGTGAATATTAAAAGAGTCGA
Northern RNA1	R1-probe	TTGGCGTAGCATCGACTCTCGGTAATAAAACGATTCGCA
Northern RNA1	R2-probe	ATTCGTCTGTTTATATAATTTTTTG
Northern 5S rRNA	5S-probe	TTAACTTCTGTGTTCGGCATGGGAACAGGTGTGACCTCC

Supplementary Figure 1, Overview of pSA564 and derived constructs.

Upper panel: Complete linear map of pSA564, with annotated open reading frames in green. Open reading frames of special interest are labelled in red.

Middle panel: Zoom of the region from position 18590 to 21937 on pSA564. The equivalent of the pSK1 replication origin has been marked in purple, although no experimental evidence is available fro pSA564.

Lower panel: Blue lines indicate the regions cloned into each family of constructs used in this study. The vectors used as backbones for the constructs are shown in black.

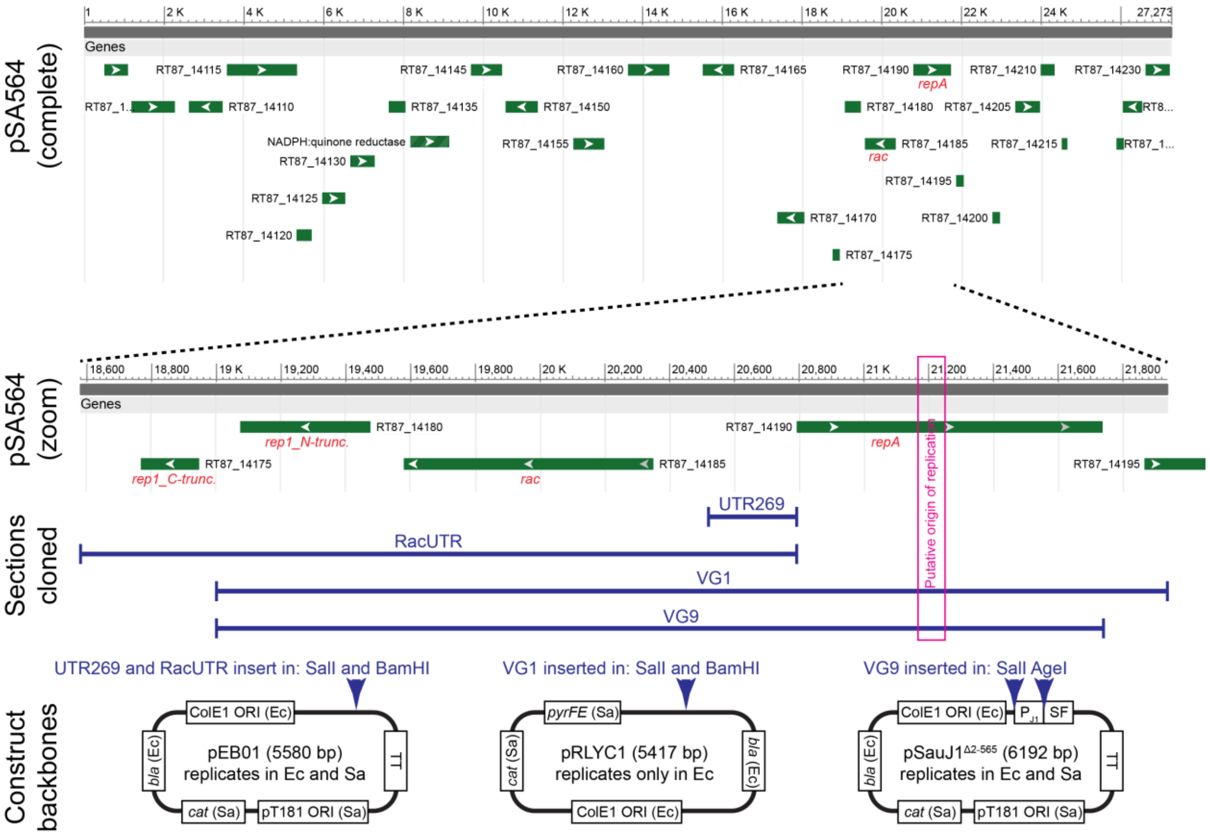

Supplementary Figure 2, full nucleotide sequences of the P_{RNA1}* and kiss* mutations

Mutated nucleotides are in red and GG^{UP} is indicated in green.

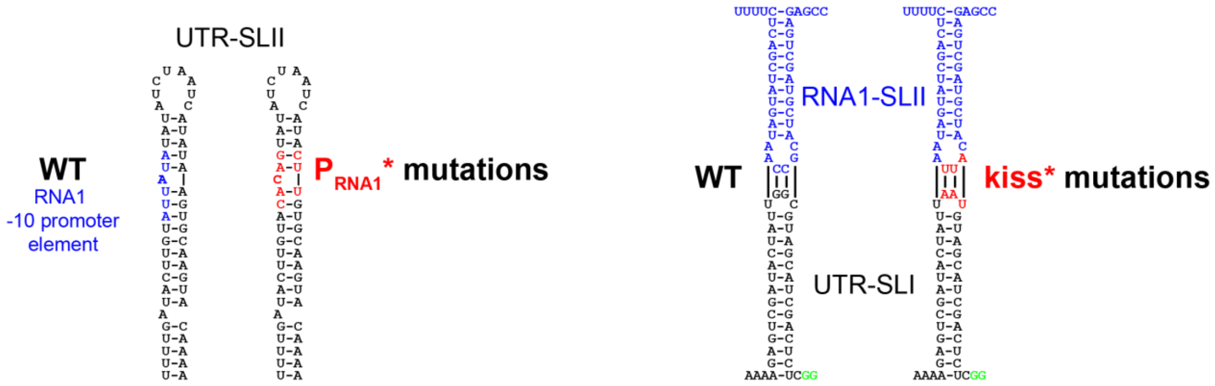

Supplementary Figure 3, Short RNA1 versions:

A) Overview of the RNA1 locus in pUTR269 and pRacUTR.

B) The RNA1 gene as well as the RNA1-18nt and RNA1-38nt nucleotide sequences, with the respective probable transcripts underlined. The -35 element, the -10 element, and the putative hairpins RNA1-SLI and RNA1-SLII are shown in bold.

C) RNA1-18nt is able to purge pSA564 from the PR01 strains, but RNA1-38nt is not. The pEB01, pUTR269 and pRacUTR are the same photos as in Figure 2, and all spots were done on the same petri dishes. MH: Mueller-Hinton medium, MHC: MH with chloramphenicol, MHP: MH with penicillin, MHCP: MH with chloramphenicol and penicillin.

D) Northern blot showing that RNA1-18nt and RNA1-38nt are both expressed. The additional full-length RNA1 band seen in the pRNA1-38nt lane comes from the pSA564 plasmid which is still present in these cells. Loading control: methylene blue staining of the membrane.

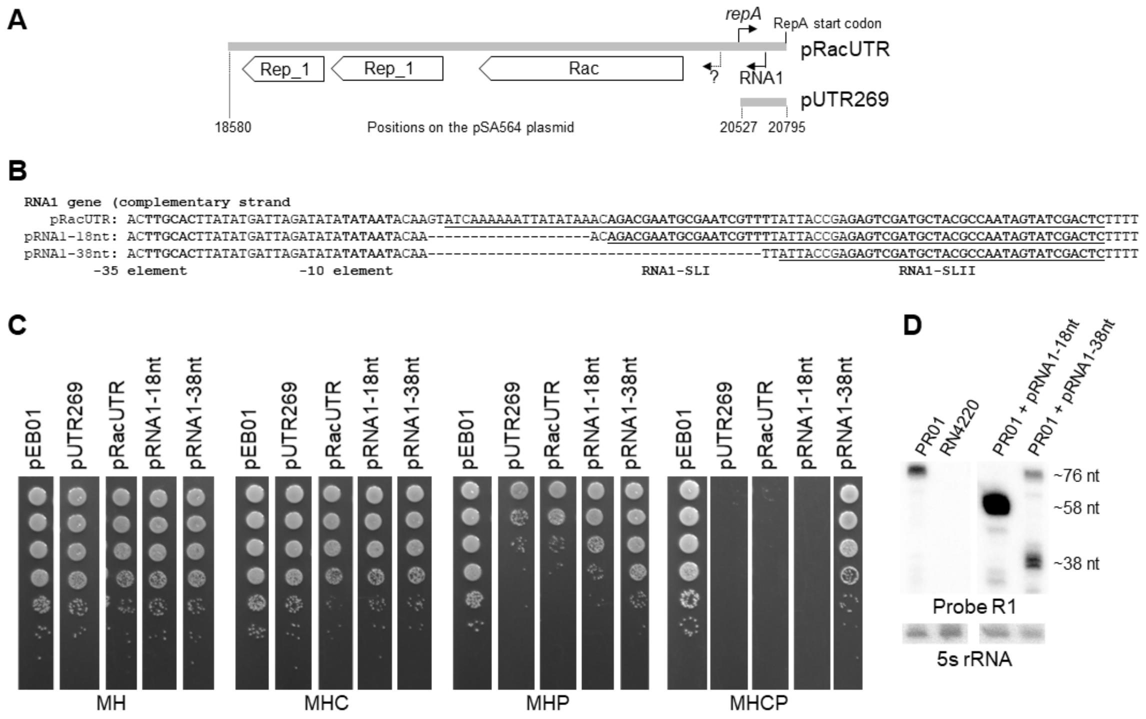

Supplement: Supplementary file 1 [file Data_Sheet_1.pdf]
